# Transmission Line Voltage Measurement Utilizing a Calibrated Suspension Grounding Voltage Sensor

**DOI:** 10.3390/s23167161

**Published:** 2023-08-14

**Authors:** Rujin Huang, Wenbin Zhang, Junyu Zhu, Xiangqi Zou, Hetao Wu, Chunguang Suo

**Affiliations:** 1 College of Science, Kunming University of Science and Technology, Kunming 650504, China; 20212111077@stu.kust.edu.cn (R.H.); zhujunyu@stu.kust.edu.cn (J.Z.); 2 College of Mechanical and Electrical Engineering, Kunming University of Science and Technology, Kunming 650504, Chinazouxiangqi8023@126.com (X.Z.); 20212203051@stu.kust.edu.cn (H.W.)

**Keywords:** voltage measurement, suspension grounding, self-calibration, internal capacitance transformation

## Abstract

The accurate voltage measurement of distribution networks is of great significance in power dispatching and fault diagnosis. Voltage sensors based on the spatial electric field effect do not require grounding, which provides the possibility for the distributed measurement of transmission line voltages. However, the divider ratio of suspension grounding voltage sensors is affected by the height between the sensor and the ground, as well as the distance between the sensor and the telegraph pole. In this paper, a self-calibration method based on internal capacitance transformation is proposed to realize the on-line calibration of suspension grounding voltage sensors. The calibration is accomplished by switching different parameters in the conditioning circuit, and the calibration process does not require power failure or known input excitation. In addition, the impact of electric fields in the other two phases of three-phase transmission lines on measurement through simulation research is quantified in this paper. In order to reduce the impact of interference electric fields, an equipotential shielding structure is designed. The circuit topology and probe prototype have been developed and testing has been conducted in laboratory conditions; the experimental results show that the maximum relative error of voltage amplitude is 1.65%, and the phase relative error is 0.94%. The measurement accuracy is not limited by the height to ground or the distance to the telegraph pole. In addition, in the application of an equipotential shielding probe, the maximum deviation of measured voltage is 0.7% with and without interference electric fields.

## 1. Introduction

With the development of smart grid technology, the characteristics of the distribution network are becoming more and more complex, and the real-time online monitoring of the operating state of the distribution network has been suggested, which has higher requirements. Real-time and accurate distributed measurement provides a new idea for the monitoring of the distribution network [1,2,3,4]. There are many current measuring devices for power systems, such as magnetic components [5,6], Hall sensors [7], Process Control Blocks (PCBs) and Roche coils [8]. Unlike voltage sensors, current sensors are not affected by ground potential, so they can be calibrated in the lab or factory and maintain high accuracy at the application site. In terms of voltage sensors, the distributed measurement of the phase voltage of overhead transmission line is always a difficult point. In voltage measurement, the most widely used voltage sensors are Power Transformers (PTs), capacitor voltage transformer (CVTs) and optical voltage sensors. The main limitations of PTs and CVTs is their high cost, complex insulation structure, large size and installation difficulties [9,10]. And these devices need to be reliably grounded, although some studies have made improvements in grounding methods [11,12]. The above disadvantages prevent them from being placed on a large scale in the distribution system. Optical voltage sensors are one of the important measurement tools for relay protection and energy monitoring in distribution networks [13,14]. However, due to their high technical requirements, poor stability and high cost, they have not been widely applied.

Miniaturization, convenience and low cost for large-area deployment are the way forward for voltage measurements [15,16]. Some scholars have already started to use the principles of space electric field and capacitance to carry out research related to voltage measurement. A commonly used voltage measurement method is the voltage measurement technology based on the principle of stray capacitance voltage division [17,18,19]. The voltage divider is formed according to the coupling stray capacitance between the induced electrode and the measured wire and the inherent capacitance of the designed sensor; the voltage measurement value of the measured wire can be obtained according to the ratio of capacitance mentioned above. Since the capacitance between the induced electrode and the measured wire needs to be determined, such sensors are usually designed to measure special scenarios, such as specially designed GIS tanks, or to measure very fast transient overvoltage (VFTO) signals without a known frequency response [20]. This measurement technology needs to be connected to the real ground; it is difficult to apply in the measurement of high voltage due to the problem of insulation.

Transmission line measurement technology is used to capture the electric field emitted by a wire through an ungrounded electric field sensor, and the voltage of the wire is reconstructed through the relationship between the sensor output signal and the transitive relation [21,22]. The advantage of this technology is that it can be measured completely without contact. However, since the capacitance of the sensor to the measured wire and the ground is difficult to obtain, complex calibration is required, which is usually accompanied by the interruption of the power system and the injection of known excitation into measured wire, so it is difficult to apply in practical engineering. To overcome this limitation, an on-line calibration technique based on system identification is developed [23,24]. This technology eliminates the influence of unknown capacitance between the probe and the wire by injecting a reference signal into the induced electrode, greatly improving measurement accuracy. However, since the transmission line is usually at least a three-phase line and the sensor needs to deal with the electric field from multiple wires, the above calibration method will be interfered with by the surrounding coupled electric field in the transmission line scene, and its calibration accuracy will be limited by the interference electric field.

In references [25,26], an array electric field sensor is used to measure the electric field at multiple locations, and an algorithm based on electric field integration is used to reconstruct the transmission line voltage. But in actual measurements, the position of the array sensor with respect to the ground and the transmission line is usually unknown, and the telegraph pole also changes the distribution of the spatial electric field. 

A suspension grounding voltage sensor based on the spatial electric field effect was proposed in reference [27]. The sensor body is electrically and mechanically connected to a single conductor. The sensor based on the space electric field effect uses air as the insulating medium, and the induced electrode in the sensor is coupled to the real ground by stray capacitance to create a capacitance divider. When used, it can be directly hung on the wire without grounding. Due to the direct use of air insulation, the measurement technology has low insulation requirements. However, in the capacitive divider formed in this way, the stray electricity to the ground is easily affected by the distance and the environment, which will affect the measurement accuracy to a certain extent. In addition, the authors do not consider the influence of other phase-line coupled electric fields.

Based on the floating voltage sensor, this paper solves the problem of the capacitance between the sensor and ground being difficult to determine and other phase electric field interferences. A self-calibration method based on internal capacitance transformation is proposed to realize the on-line calibration of the voltage ratio in actual measurement. At the same time, an equipotential shielding structure is designed to reduce the interference of other phases without grounding shielding. Firstly, the basic principle of a suspension grounding voltage sensor based on the spatial electric field effect is introduced, and the affecting factors of the coupling capacitance between the probe and the earth are analyzed. Then, the selected parameters are optimized via error analysis. A scheme to solve the uncertainty of capacitance to ground is proposed and a circuit is designed. Then, the selected parameters of circuit parameters are optimized via error analysis. Through simulation, the influence of other two-phase electric fields on voltage measurement is quantified. In order to reduce the influence of interfering electric fields, an equipotential shielding structure is designed. On this basis, the sensor probe and circuit topology are developed. Finally, the sensor prototype is tested for amplitude and phase accuracy, scene adaptability and anti-interference ability. The experimental results demonstrate the feasibility of this method, which may provide a new direction for the research and development of transmission line voltage measurement methods. Finally, the shortcomings of this measurement method and the future plans are discussed.

## 2. Principle of Voltage Measurement

### 2.1. Suspension Grounding Voltage Sensor Based on Spatial Electric Field Effect

**Figure 1:**
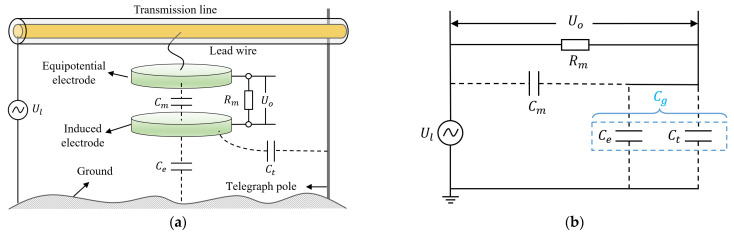
Suspension grounding voltage sensor based on spatial electric field effect. (**a**) Schematic diagram; (**b**) equivalent circuit diagram.

The measurement principle of a floating voltage sensor based on the spatial electric field effect is shown in Figure 1a. Including an equipotential electrode and induced electrode, the equipotential electrode is connected to the transmission line through lead wire, and the capacitance formed by the induced electrode and the ground is $C_{e}$. The equipotential electrode is connected to the induced electrode through a structural capacitance $C_{m}$ and a sampling resistor Rm, and the voltage across the sampling resistor is $U_{o}$. The voltage of the transmission line is $U_{l}$. The equivalent circuit according to Figure 1a is shown in Figure 1b. The transfer function obtained from the equivalent circuit diagram is shown in Equation (1) [28], where $C_{g}=C_{e}+C_{t}$.

(1) $$\frac{U_{o}(s)}{U_{l}(s)}=\frac{sR_{m}C_{g}}{1+sR_{m}(C_{m}+C_{g})}$$

The frequency response of $U_{o}$ is the frequency response of a high-pass filter with a cutoff frequency of $f_{1}=1/2\pi R_{m}\left(C_{m}+C_{g}\right)$ [29]. If the operating frequency of the voltage sensor is much greater than $f_{1}$, Equation (1) can be simplified as:

(2) $$\frac{U_{o}}{U_{l}}=\frac{C_{g}}{C_{m}+C_{g}}$$

If the coupling capacitance $C_{m}$, $C_{g}$ and the sensor output voltage signal $U_{o}$ are known, the voltage $U_{l}$ of the transmission line can be reconstructed using Equation (2). The capacitance $C_{m}$ is a structural capacitance that can be obtained through digital bridges or simulations, and the sensor output voltage $U_{o}$ can be obtained through data acquisition instruments. The coupling capacitance $C_{g}$ is affected by the height to ground, the specifications of the telegraph pole and the distance between the sensor and the telegraph pole. In actual measurement, $C_{g}$ is difficult to determine.

**Figure 2:**
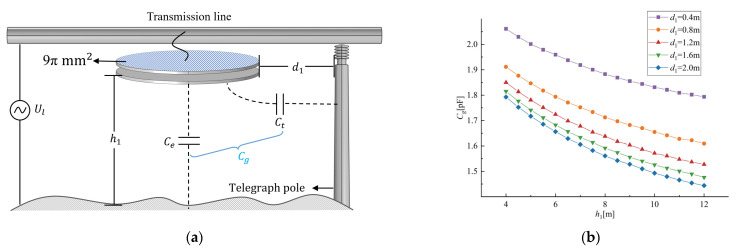
Simulation of the influence of sensors on ground height and telegraph pole distance. (**a**) Simulated models and parameters; (**b**) the influence of different ground heights $h_{1}$ and telegraph pole distances $d_{1}$ on $C_{g}$.

In order to quantify the sensitivity of $C_{g}$, the finite element simulation software COMSOL was used to calculate the size of $C_{g}$ at different ground heights and distances from the telegraph pole. The simulation parameters are shown in Figure 2a. The distance $d_{1}$ between the probe and the telegraph pole increased from 0.4 m to 2.0 m in steps of 0.4 m, and the distance $h_{1}$ between the probe and the ground increased from 4 m to 12 m in steps of 0.5 m. The simulation results obtained are shown in Figure 2b. According to the simulation results, the deviation in $C_{g}$ between $d_{1}$ = 0.4 m and $d_{1}$ = 0.8 m at $h_{1}$ = 4 m is 7.3%, and the deviation in $C_{g}$ between $d_{1}$ = 0.4 m and $d_{1}$ = 2 m at $h_{1}$ = 4 m is 19.5%. The deviation in $C_{g}$ between $h_{1}$ = 4 m and $h_{1}$ = 5 m at $d_{1}$ = 0.4 m is 2.96%, and the deviation in $C_{g}$ between $h_{1}$ = 4 m and $h_{1}$ = 12 m at $d_{1}$ = 0.4 m is 12.9%. It can be seen that the capacitance $C_{g}$ will be affected by the installation position of the sensor relative to the telegraph pole and the ground. From Equation (1), it can be seen that replacing $C_{g}$ with a fixed coefficient will result in a measurement error proportional to the offset rate of the capacitor.

### 2.2. Self-Calibration Method Based on Internal Capacitance Transformation

**Figure 3:**
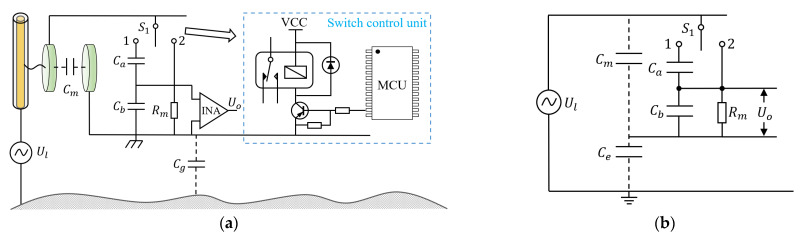
Calibration scheme based on internal capacitance transformation. (**a**) Schematic diagram; (**b**) equivalent circuit diagram.

A self-calibration method based on internal capacitance transformation is proposed to achieve the acquisition of $C_{g}$ and voltage reconstruction. The schematic diagram is shown in Figure 3a. On the basis of Figure 1a, the schematic diagram incorporates a lumped capacitor $C_{a}$ and a single pole double throw switch $S_{1}$ at both ends of the structural capacitance $C_{m}$, which is a relay switch controlled by a microcontroller (MCU). The relationship between $C_{a}$ and $C_{b}$ is a series connection. $U_{o1}$ and $U_{o2}$ represent the output voltage of the instrumental amplifier (INA) when the switch is turned to 1 and 2, respectively.

The equivalent circuit diagram of Figure 3a is shown in Figure 3b. When the switch is turned to 1, the transfer function is shown in Equation (3), where $k=C_{a}/C_{a}+C_{b}$.

(3) $$\frac{U_{o1}}{U_{l}}=\frac{sR_{m}C_{g}}{1+sR_{m}(kC_{b}+C_{m}+C_{g})}$$

When $\omega >\omega_{h1}=\frac{1}{R_{m}\left(kC_{b}+C_{m}+C_{g}\right)}$, the above transfer function can be simplified as:

(4) $$\frac{U_{o1}}{U_{l}}=\frac{kC_{g}}{kC_{b}+C_{m}+C_{g}}$$

When the switch is turned to 2, the transfer function can be expressed as:

(5) $$\frac{U_{o2}(s)}{U_{i}(s)}=\frac{skR_{m}C_{g}}{1+sR_{m}\left(C_{b}+C_{m}+C_{g}\right)}$$

When $\omega >\omega_{h2}=\frac{1}{R_{m}\left(C_{b}+C_{m}+C_{g}\right)}$, the above transfer function can be simplified as:

(6) $$\frac{U_{o2}}{U_{l}}=\frac{C_{g}}{C_{b}+C_{m}+C_{g}}$$

By eliminating the simultaneous Equations (4) and (6), the following can be obtained:

(7) $$C_{g}=\frac{U_{o2}k(C_{b}+C_{m})-U_{o1}(kC_{b}+C_{m})}{U_{o1}-kU_{o2}}$$

(8) $$U_{l}=\frac{U_{o1}U_{o2}(kC_{b}-C_{b})}{U_{o1}\left(kC_{b}+C_{m}\right)-U_{o2}k\left(C_{b}+C_{m}\right)}$$

By controlling whether the capacitor $C_{a}$ is connected to the circuit or not, two different transfer relations and output voltages can be obtained. The unknown capacitance $C_{g}$ can be obtained by means of Equation (7). Further, the measured voltage $U_{l}$ can be obtained via Equation (8).

## 3. Topology Circuit and Shielded Probe Design

### 3.1. Analysis of Calibration Accuracy Impact and Parameter Optimization

The selection of capacitance parameters is the key to improving voltage calibration accuracy. When there is an error in the output voltage of the sensor substituted in actual measurement, using Equation (8) for calibration may cause the error to expand infinitely. Reference [23] conducted simulation calculations and analyses on the calibration model in [30] and found that when there are almost equal subtractions in the calibration formula, the error will propagate widely. The use of capacitors with an accuracy of 0.01% will result in an error of −15.5% in the calibration voltage. Fortunately, the propagation of errors can be reduced through parameter optimization.

**Figure 4:**
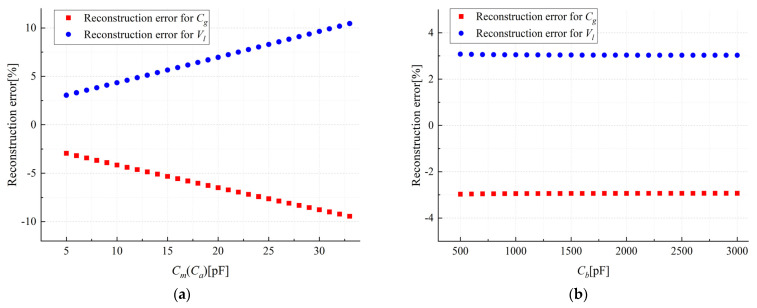
(**a**) Influence of $C_{m}$($C_{a}$) on the reconstructed voltage; (**b**) influence of $C_{b}$ on the reconstructed voltage.

In order to explore the optimal parameter range, the parametric scanning of the equivalent circuit model is simulated. Assuming the model in Figure 3b is a pure capacitor network, the initial values of $C_{m}$, $C_{a}$, $C_{b}$ and $C_{g}$ are set to 20 pF, 5 pF, 1 nF and 2 pF, respectively. The variation range is shown in the horizontal axis in Figure 4, and all parameters except for variables remain unchanged as the initial parameters. We changed the output voltage $U_{o1}$ and $U_{o2}$ to (1 − 0.005)$U_{o1}$ and (1 − 0.005)$U_{o2}$, respectively, to simulate a 0.5% error in the output of the sensor. We substituted the output voltage with errors into Equation (8) to obtain the calibration voltage and calculated the relative error between the calibration voltage and the actual voltage. The curves of each capacitor parameter and relative error are shown in Figure 4.

**Figure 5:**
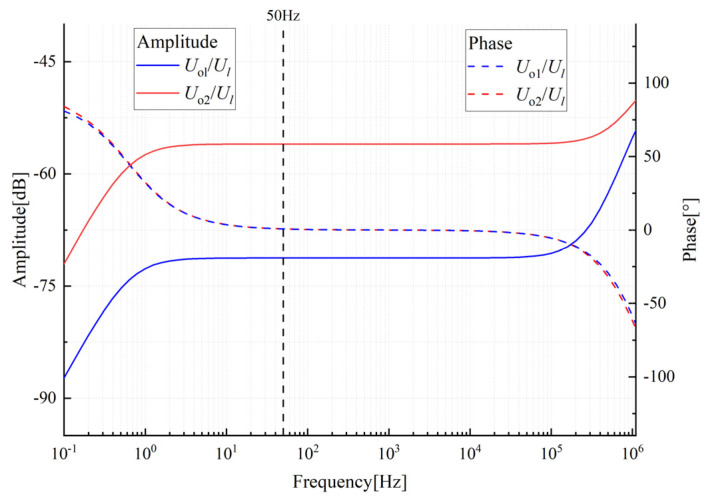
Voltage sensor circuit amplitude- and phase-frequency characteristics.

From Figure 4, it can be seen that $C_{m}$ and $C_{a}$ are negatively correlated with calibration accuracy. Therefore, reducing $C_{m}$ and $C_{a}$ can improve the calibration accuracy. The impact of $C_{b}$ on accuracy can be ignored, as it only needs to meet a certain voltage ratio to reduce the output signal to the collectable voltage range. In the subsequent probe and circuit design, the capacitors and resistors are set as shown in Table 1. The instrumentation amplifier used is AD8220ARMZ, and the gain is set to 1. The amplitude frequency characteristic curve and phase frequency characteristic curve of this circuit are shown in Figure 5. From the figure, it can be seen that there is a flat frequency response in the range of 20 Hz to 50 kHz, and the sensor output voltage is independent of phase and frequency. When measuring power frequency voltage, Equation (8) can be used for voltage reconstruction.

### 3.2. Equipotential Shielding Probe

**Figure 6:**
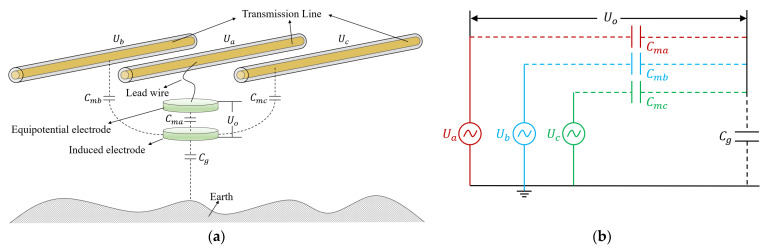
(**a**) Transmission line measured model; (**b**) equivalent circuit diagram.

In actual transmission line measurement, there are at least two adjacent transmission lines besides the measured wire. At the same time, the electric field of adjacent lines will also be coupled to the induced electrode through capacitance, as shown in Figure 6a. Among them, the voltages on transmission lines phase-A, phase-B, and phase-C are $U_{a}$, $U_{b}$, and $U_{c}$. Assuming that the sensor is used to measure the voltage of phase- A, the equipotential electrode is connected to phase-A to form an equipotential. The capacitance formed by the induced electrode and phase-A, phase-B and phase-C is $C_{ma}$, $C_{mb}$ and $C_{mc}$, respectively. The equivalent circuit according to Figure 6a is shown in Figure 6b, and the transfer function obtained from this equivalent circuit diagram is as follows:

(9) $$U_{o}=U_{a}-(\frac{C_{ma}U_{a}}{C_{g}+C_{ma}}+\frac{C_{mb}U_{b}}{C_{g}+C_{mb}}+\frac{C_{mc}U_{c}}{C_{g}+C_{mc}})$$

**Figure 7:**
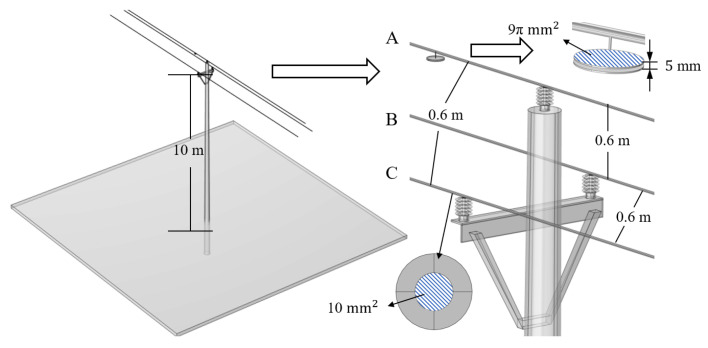
Simulation model of the suspension grounding voltage sensor affected by the other two phases.

**Figure 8:**
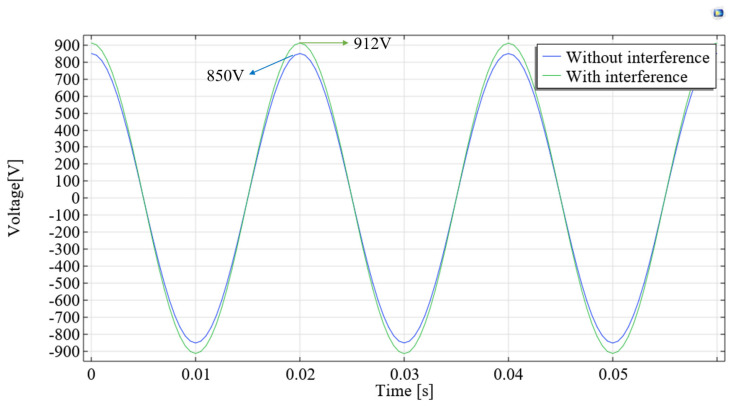
Simulation results for steady-state waveforms of flat structure.

It can be seen from Equation (9) that when one phase of a three-phase transmission line is measured using a suspension grounding voltage sensor based on the electric field effect, the electric field generated by the remaining two phases will be coupled to the sensor. Due to the fact that all transmission lines have the same frequency of electric fields, the signals captured by the sensor cannot be separated. In order to explore the influence of other adjacent lines, the finite element simulation was used to quantify it. The parameters in the simulation model are shown in Figure 7. The simulation results are shown in Figure 8. When only phase-A has a voltage amplitude of 10 kV, the potential of the induced electrode is 850 V. When the voltage amplitude of phase-A, phase-B and phase-C is 10 kV and the phase difference is 120°, the induced electrode potential is 912 V, with a deviation of 6.8%. It is necessary to design shielding probes to reduce the interference of adjacent lines.

**Figure 9:**
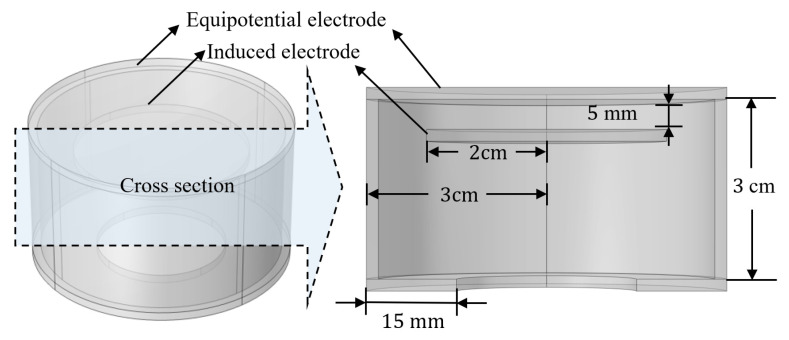
Structure and parameters of equipotential shielded probe.

**Figure 10:**
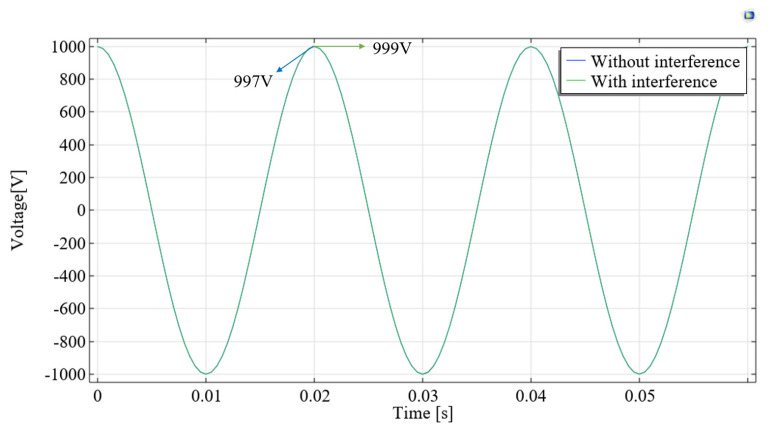
Voltage of induced electrode under equipotential shielded probe application.

As can be seen from Equation (9), by increasing $C_{ma}$ and decreasing $C_{mb}$, $C_{mc}$ can enhance the anti-interference ability of the sensor. However, in the analysis of Section 3.1, increasing $C_{ma}$ will result in a decrease in calibration accuracy. Therefore, an equipotential shielding structure that significantly reduces the capacitance $C_{mb}$ and $C_{mc}$ without significantly increasing $C_{ma}$ is designed in this paper. The structural schematic diagram and parameters are shown in Figure 9. To verify its performance, the flat plate probe in Figure 7 was replaced with the equipotential shielding probe. Figure 10 shows the simulation results, which show that the potential of the induced electrode with and without interfering wires is 997 V and 999 V, respectively, with a difference of 0.2%. Table 2 shows the inherent capacitance values of two types of probes and the comparison of capacitance parameters for the adjacent line. Compared to the flat structure, the capacitance $C_{ma}$ increases by 1.37 times, and the capacitance of $C_{mb}$ and $C_{mc}$ decreases by 78 times.

### 3.3. Control Steps and Measurement Process

**Figure 11:**
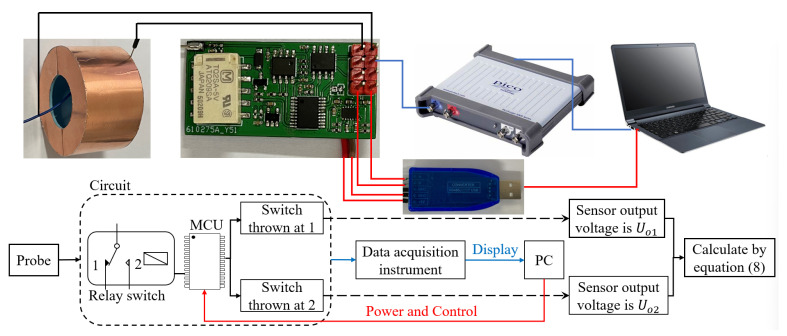
Measurement system and procedure.

The measurement framework used in this article is shown in Figure 11. The induction probe is connected to the conditioning circuit, which includes a switching capacitor, relay, instrumentation amplifier, single chip microcontroller and +5 V conversion ±5 V module power. The conditioning circuit is connected to the data acquisition device to read the output signal and display it in real time on a personal computer. At the same time, the conditioning circuit is connected to a personal computer through a converter to provide power and switch control commands for the conditioning circuit. When the switch is controlled to 1, the sensor output signal is marked as $U_{o1}$. When the switch is controlled to 2, the sensor output signal is marked as $U_{o2}$. The obtained output value and known parameter values are used to reconstruct the voltage according to Equation (8).

## 4. Experimental Testing and Result Analysis

### 4.1. Establishment of Experimental Platform

**Figure 12:**
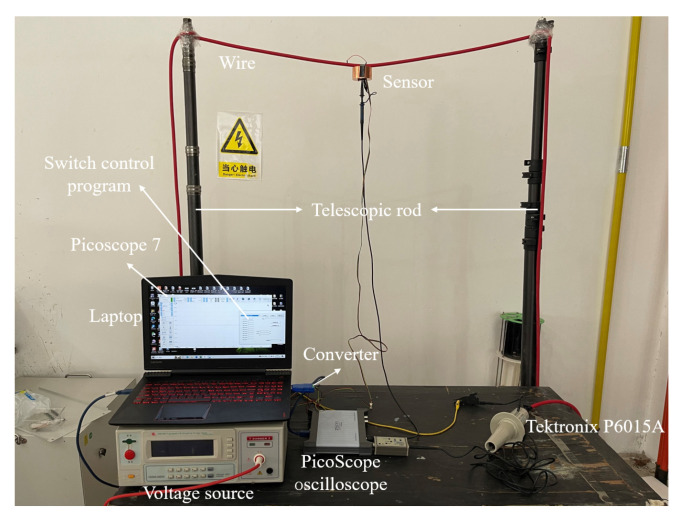
Experimental platform.

In order to verify the feasibility of the calibration method for suspension grounding voltage sensor based on internal capacitance transformation and the performance of the equipotential anti-interference probe, a testing platform was built in the laboratory, and the testing site is shown in Figure 12. The AC voltage source is CX9914BX, from Changsheng, which can provide 30–300 Hz, 0–5 kVrms AC voltage output. Its output is connected to the tested wire with a length of 20 m. The data acquisition instrument is a Picoscope 5000D PC oscilloscope from Pico Technology (Cambridgeshire, UK), with a resolution of 16 bits and a sampling rate of 62.5 MS/s. The oscilloscope is connected to a mobile personal computer (PC) through a Universal Serial Bus (USB), and the output signal collected is displayed through PicoScope7 software (7.0.116). The mobile PC is battery-powered, making the measurement system completely isolated from the actual ground. The voltage and phase of the measured wire are calibrated by the Tektronix 6015A probe, which is also connected to the oscilloscope and displayed on PicoScope7.

During the experiment, the induced probe is placed below the measured wire, and the equipotential electrode of the probe is connected to measured wire. The measured wire is mounted on two stretchable poles, with a maximum stretchable distance of 8 m. The Tektronix 6015A high-voltage probe is connected at both ends of the measured wire and the actual ground.

### 4.2. Amplitude and Phase Accuracy Testing

The following steps are carried out when the measured wire is located at a height of 3 m. Perform the following experimental steps on the established experimental platform. In the first step of testing, turn switch $S_{1}$ to 1 and record the output $U_{o1}$ of the signal processing circuit observed from the oscilloscope. In the second step of testing, turn switch $S_{1}$ to 2 and record the output $U_{o2}$ of the conditioning circuit observed from the oscilloscope and the Tektronix 6015A calibrated application voltage. The output range of the AC source increases from 1000 $V_{rms}$ to 5000 $V_{rms}$ in steps of 200 $V_{rms}$. Repeat the above steps to complete the experiment. The results of the recorded values and reconstructed voltage $U_{r}$ via Equation (8) are shown in Table 3.

**Figure 13:**
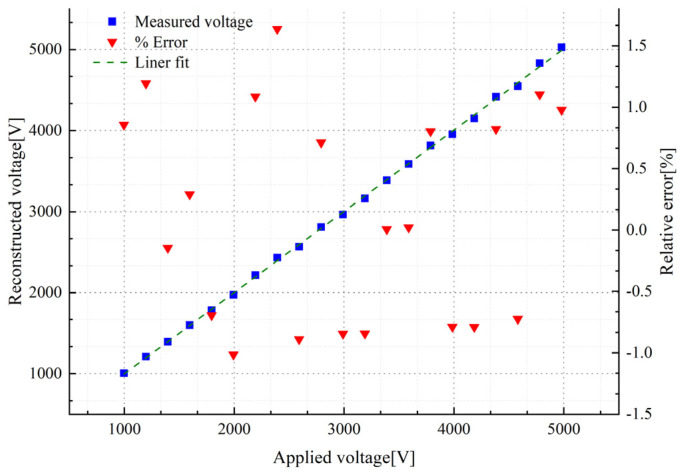
Error characteristics of actual output voltage and reconstructed voltage.

**Figure 14:**
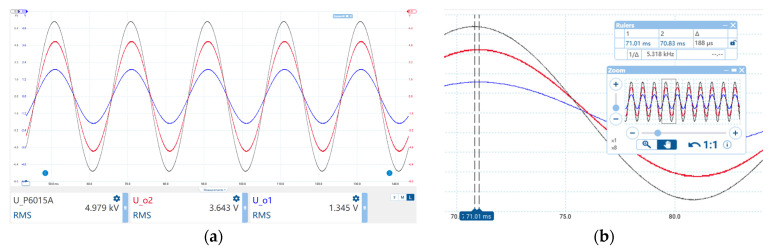
Display waveform on Picoscope7 when the output voltage is 5000 V. (**a**) The output waveform of the Tektronix P6015A and the sensor output waveform $U_{o1}$, $U_{o2}$; (**b**) phase comparison details.

Figure 13 shows the fitting curve and relative error characteristics between the reconstructed voltage $U_{r}$ and the calibrated voltage $U_{P6015A}$, and the results show that the maximum relative error of the amplitude is 1.65%. Figure 14a shows the output waveform of Tektronix P6015A and the sensor output waveforms $U_{o1}$ and $U_{o2}$ when the output voltage of the voltage source is 5000 $V_{rms}$. Figure 14b shows the phase comparison details, and the results show that the relative error of the phase is $\frac{\Delta t}{T}\cdot 100\%=\frac{188\mathrm{us}}{0.02s}=0.94\%$.

### 4.3. Scenario Adaptability Testing

**Figure 15:**
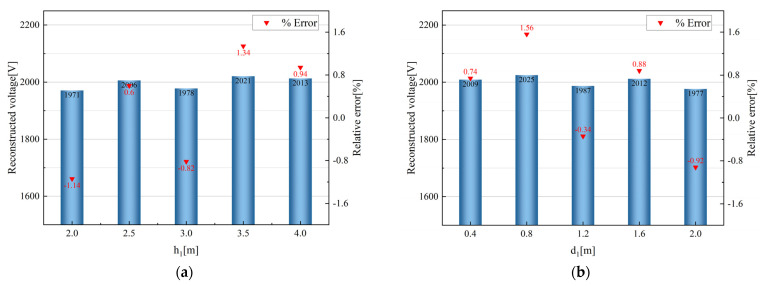
(**a**) Measured voltage and error characteristics with different $h_{1}$; (**b**) measured voltage and error characteristics with different $d_{1}$.

We wanted to verify that the measurement method proposed in this paper can be measured in different scenarios and the measurement accuracy is not affected by the measurement scenario. On the experimental platform shown in Figure 12, different tests of ground height and tower distance were carried out. By changing the distance of the telescopic rod to change the ground height $h_{1}$, $h_{1}$ increases from 2 m to 4 m in steps of 0.5 m, while maintaining $d_{1}$ at 1.2 m during this process. By changing the distance of the experimental platform and the wall to simulate the different distance $d_{1}$ between the sensor and the telegraph pole, $d_{1}$ is increased from 0.4 m to 2 m in steps of 0.4 m, while maintaining $h_{1}$ at 3 m during this process. The experimental results are shown in Figure 15, with a maximum percentage error of 1.34% with different $h_{1}$ and a maximum percentage error of 1.56% with different $d_{1}$. When the voltage of the unknown wire is reconstructed according to Equation (8), the measurement accuracy does not change significantly with changes in height to ground or distance to telegraph pole.

### 4.4. Anti Interference Achievement Test

**Figure 16:**
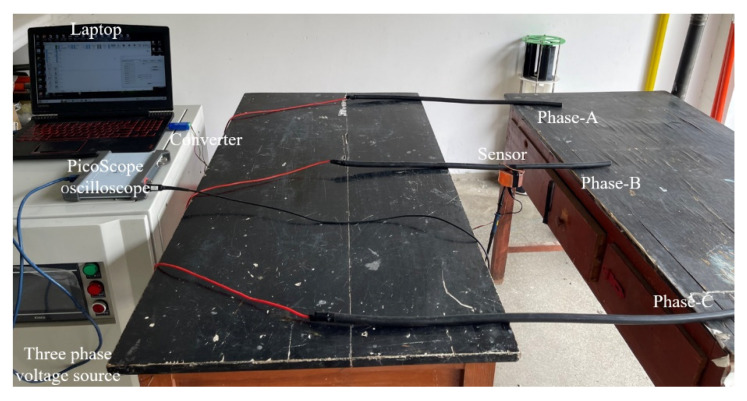
Anti-interference capability test platform.

To verify the shielding effect of the equipotential probe, experimental tests were conducted as shown in Figure 16. Compared with the experimental platform in Figure 12, replace the single-phase voltage source with the three-phase voltage source ANB13-1KA, which can provide 40–300 Hz, 0–300 $V_{rms}$ three-phase voltage output. In this test, in order to measure low voltage, the capacitor $C_{b}$ is replaced from 3 nF to 1 nF. The three-phase voltage output is connected to three cables 60 cm apart. The following experiments were conducted on this experimental platform. In the first step of testing, phase-A, phase-B and phase-C are set to the same voltage, with a phase difference of 120°. In the second test, the voltage output is only in phase-B. The output voltages are set to 100 V, 200 V and 300 V, and the reconstructed voltages and relative errors with interfered and without interfered voltage are obtained, as shown in Table 4.

As can be seen from Table 4, the sensor output voltages $U_{o1}$, $U_{o2}$ and reconstructed voltage increase slightly in the presence of the interference voltage compared to the absence of the interference voltage, but the increase is small. The deviations in the reconstructed voltage are 0.7%, 0.67% and 0.69%, respectively. It shows that the equipotential shielding has a good anti-interference property and can provide conditions for the voltage measurement of transmission lines.

## 5. Conclusions

The main work of this article is as follows: (1) In order to solve the problem that the capacitance between the sensor and ground is difficult to determine, a calibration method based on internal capacitance transformation is proposed to achieve the calibration of suspension grounding sensors. (2) We carried out theoretical research and transfer function analysis on the proposed method, analyzed the influence of calibration accuracy on system parameters through simulation and provided parameter design principles. According to this design index, the topology of capacitor conversion circuit was formulated. (3) The influence of the other two-phase electric fields on the measurement of three-phase transmission lines was quantified through simulation research. In order to reduce the impact of adjacent transmission lines, an equipotential shielding structure was designed. (4) The calibration accuracy test was carried out with the self-made sensor prototype under the power frequency voltage 1–5 kV, and the results showed that the maximum relative error is 1.65% and the relative error of phase is 0.94%. Then, scenario adaptability testing was conducted. The results show that in different measurement scenario experiments, the maximum relative error is 1.56%, and the measurement accuracy is not limited by the height of the sensor to the ground or the distance to the pole. Finally, the anti-interference achievement test is carried out. The experimental data show that the maximum deviation of the reconstructed voltage is 0.7% when there is no interfering electric field. This shows that these potential shielding probes have good shielding ability to the external coupled electric field.

It can be seen from the experimental results that the self-calibration method based on internal capacitance transformation can realize the calibration of suspension grounding voltage sensors in different measurement scenarios. The developed equipotential shielding probe can greatly reduce the interference of external electric fields. It is of practical significance to extend the suspension grounding voltage measurement based on the space electric field effect. The structure in the manuscript is only intended for the verification of a suspension grounding voltage sensor. The device may be affected by the accumulation of rainwater or dust. In future practical applications, the sensor will be enclosed in an enclosed space to prevent rain and dust from affecting the interior of the sensor.

## Figures and Tables

**Table 1:** The value of the selected parameter.

Parameters	Description	Value
$C_{m}$	Capacitance between induced electrode and equipotential electrode	5 pF
$C_{a}$	Transformation capacitor	5 pF
$C_{b}$	Sampling capacitor	3 nF
$R_{m}$	Sampling resistor	50 MΩ

**Table 2:** Comparison of capacitance in plate structure and equipotential shielding structure.

Parameters	Description	Values	
		Flat Structure	Equipotential Shielding Structure
$C_{ma}$ [pF]	Capacitance between induced electrode and equipotential electrode	3.39	4.64
$C_{mb}$ [pF]	Capacitance between induced electrode and phase-B conductor	0.125	0.0016
$C_{mc}$ [pF]	Capacitance between induced electrode and phase-C conductor	0.125	0.0016

**Table 3:** Accuracy test results.

$U_{P6015A}/V$	$U_{o1}/\mathrm{mV}$	$U_{o2}/\mathrm{mV}$	$U_{r}/V$
997	269.2	616.2	1000
1197	323.8	738.2	1205
1396	376.0	861.7	1388
1595	430.5	984.0	1611
1795	483.2	1107	1787
1994	535.9	1229	1972
2193	591.8	1353	2213
2392	646.0	1475	2431
2591	697.3	1598	2575
2791	753.1	1722	2813
2990	803.7	1843	2958
3189	858.3	1967	3169
3388	914.1	2093	3390
3587	966.7	2214	3581
3786	1022	2337	3816
3985	1073	2460	3954
4184	1127	2584	4151
4383	1183	2705	4419
4581	1234	2829	4548
4780	1290	2948	4833
4979	1345	3075	5028

**Table 4:** Anti-interference test results.

Applied Voltage [V]	With/Without Interference	$U_{o1}\;[\mathrm{mV}]$	$U_{o2}\;[\mathrm{mV}]$	Reconstructed Voltage [V]	% Deviation
200	Without	204.3	489.5	199.7	0.7
	With	205.5	492.4	201.3	
250	Without	254.3	609.4	249.2	0.67
	With	256.1	613.7	250.9	
300	Without	307.1	735.2	301.3	0.69
	With	309.2	740.3	303.4	

## Data Availability

Not applicable.

## References

[1] Wu S., Han J., Cai C., Wang Q. (2023). Topology identification method of low-voltage distribution network based on measurement data of IOT devices. Energy Rep..

[2] Pegoraro P.A., Brady K., Castello P., Muscas C., von Meier A. (2019). Compensation of systematic measurement errors in a pmu-based monitoring system for electric distribution grids. IEEE Trans. Instrum. Meas..

[3] Liu X., Zhang X. (2015). Distributed voltage security monitoring in large power systems using synchrophasors. IEEE Trans. Smart Grid.

[4] Lee H., Srivastava A.K., Krishnan V.V., Niddodi S., Bakken D.E. (2021). Decentralized voltage stability monitoring and control with distributed computing coordination. IEEE Syst. J..

[5] Yan P., Zhang W., Yang L., Zhang W., Yu H., Huang R., Zhu J., Liu X. (2023). Online calibration study of non-contact current sensors for three-phase four-wire power cables. Sensors.

[6] Li W., Tan X., Ao G., Xu X., Zhang W., Cheng K. (2023). Magnetic sensor-based switching cabinet busbar current measurement method. Integr. Ferroelectr..

[7] Crescentini M., Syeda S.F., Gibiino G.P. (2021). Hall-effect current sensors: Principles of operation and implementation techniques. IEEE Sens. J..

[8] Xu Q., Feng Y., Guo P., Mo N., Xu B., Qing Z., Chen Y., Luo A. (2023). Design of pcb rogowski coil current sensor with low droop distortion. IEEE Trans. Power Electron..

[9] Xuan H., Wen C., Song Y., Wei X. (2020). Fault detection and analysis of capacitive components of capacitive voltage transformer. E3S Web Conf..

[10] Zhang Y., Zhang C., Li H., Chen Q. (2022). An online detection method for capacitor voltage transformer with excessive measurement error based on multi-source heterogeneous data fusion. Measurement.

[11] Ahmad R., Kassas M., Ahmed C.B., Khan F., Khan S., Jamal A., Ullah I. (2021). Application of mineral compounds for a high-voltage portable grounding system: An experimental study. Electronics.

[12] Ahmad R., Khan F., Jamal A., Khan S., Ali S., Horoub M.M., Albalasie A. Simulation and breakdown characteristics of china clay and silica sand for improved grounding system. Proceedings of the 2020 International Conference on Electrical, Communication, and Computer Engineering (ICECCE).

[13] Zheng W., Li H., Feng C., Wen C., Zeng X. (2022). Design of novel resonant optical voltage sensor based on pockels effect. IEEE Sens. J..

[14] Fusiek G., Niewczas P. (2022). Construction and evaluation of an optical medium voltage transducer module aimed at a 132 kV optical voltage sensor for wampac systems. Sensors.

[15] Zhenhua L.I., Shuang Z., Weizhong H.U., Zhenxing L.I. (2018). Review of the study of high voltage measurement technology. High Volt. Eng..

[16] Liu X., He W., Guo P., Yang Y., Guo T., Xu Z. (2022). Semi-contactless power measurement method for single-phase enclosed two-wire residential entrance lines. IEEE Trans. Instrum. Meas..

[17] Martins A.V., Bacurau R.M., dos Santos A.D., Ferreira E.C. (2019). Nonintrusive energy meter for nontechnical losses identification. IEEE Trans. Instrum. Meas..

[18] Suo C., Huang R., Zhou G., Zhang W., Wang Y., He M. (2023). Self-calibration sensor for contactless voltage measurement based on dynamic capacitance. Sensors.

[19] Yang P., Wen X., Chu Z., Ni X., Peng C. (2021). Non-intrusive dc voltage measurement based on resonant electric field microsensors. J. Micromech. Microeng..

[20] Wang H., Zhang X., Han X., Sun Y., Chen H., Li J. (2022). A novel composite sensor for overvoltage and uhf partial discharge measurement in gis. IEEE Trans. Power Deliv..

[21] Can G., Jingang W., Jie Y., Hu P., Jun M. (2016). Experiment and simulation of d-dot voltage probe based on inverse problem of electric field. Trans. China Electrotech. Soc..

[22] Lawrence D., Donnal J.S., Leeb S., He Y. (2016). Non-contact measurement of line voltage. IEEE Sens. J..

[23] Haberman M.A., Spinelli E.M. (2019). A noncontact voltage measurement system for power-line voltage waveforms. IEEE Trans. Instrum. Meas..

[24] Shenil P., George B. (2020). Nonintrusive ac voltage measurement unit utilizing the capacitive coupling to the power system ground. IEEE Trans. Instrum. Meas..

[25] Wang J., Zhao Y., Li W., Zeng X., Tang J., Wang Y., Deng X. (2018). Research on transmission line voltage measurement method of d-dot sensor based on gaussian integral. Sensors.

[26] Wang J., Yan X., Li X., Liao J., Tao Y. (2021). Method and experimental study of transmission line voltage measurement based on a gauss-type integral algorithm. Trans. China Electrotech. Soc..

[27] Yang L., Long W., Zhang W., Yan P., Zhou Y., Li J. (2023). Transmission line voltage calibration-free measurement method. Electronics.

[28] Lim S.W., Cho C., Jin Y.S., Kim Y.B., Roh Y. (2013). Design and test of an electric field sensor for the measurement of high-voltage nanosecond pulses. IEEE Trans. Plasma Sci..

[29] Bobowski J.S., Ferdous M.S., Johnson T. (2014). Calibrated single-contact voltage sensor for high-voltage monitoring applications. IEEE Trans. Instrum. Meas..

[30] Ringsrud P.A., Huber C.N., Gallavan M.F. (2018). Non-Contact Voltage Measurement. U.S. Patent.

